# Nonbacterial Thrombotic Endocarditis of the Mitral Valve With Echocardiographic Appearances Mimicking a Papillary Fibroelastoma in a Middle-Aged Female Patient

**DOI:** 10.7759/cureus.37540

**Published:** 2023-04-13

**Authors:** Jonathan Kovacs, Abdallah Khashan, Sadat Kasanga, Shakeel Yousaf, Aaron Feingold

**Affiliations:** 1 Internal Medicine, Raritan Bay Medical Center, Perth Amboy, USA; 2 Cardiology, Raritan Bay Medical Center, Perth Amboy, USA

**Keywords:** valvular mass, cardiac mass tumor, papillary fibroelastoma, stroke, nonbacterial thrombotic endocarditis (ntbe)

## Abstract

Papillary fibroelastoma (PFE) and nonbacterial thrombotic endocarditis (NBTE) account for <1% of all cardioembolic strokes. When there is no evidence of infection, and an exophytic valve lesion is seen on echocardiography, PFE may be an initial imaging diagnosis. NBTE, or Libman-Sacks endocarditis, is a rare entity and can present with varied imaging findings. This report presents a case of embolic stroke and NBTE mimicking a PFE. We discuss a 49-year-old female with a past medical history of diabetes mellitus who presented with headache and right-hand numbness. The initial CT head was negative and the MRI brain showed multiple infarcts in the watershed areas where anterior and posterior brain circulation meet and overlap. A transesophageal echocardiogram (TEE) showed a left ventricle (LV) mass initially diagnosed as PFE. The patient was started on aspirin only with no anticoagulation since we thought the stroke was related to an embolus from a tumor, not a thrombus. The patient underwent surgery but the pathology report revealed a diagnosis of organizing thrombus with abundant neutrophilic infiltration and no neoplastic proliferation. This case report highlights the importance of a comprehensive evaluation of valvular masses and the diagnostic approaches currently available to help clinicians differentiate between various causes of embolic stroke like PFE, bacterial endocarditis, and NBTE. Early differentiation is critical because it can affect the treatment and outcome. This report shows that echocardiography of endocardial and valvular lesions may provide a differential diagnosis, but a definitive diagnosis requires microbiology and histopathology. Advanced imaging techniques such as cardiac CT or cardiac MRI may assist in identifying select cases that are at lower risk for subsequent embolic events, in which surgical intervention may safely be avoided.

## Introduction

Papillary fibroelastoma (PFE) and nonbacterial thrombotic endocarditis (NBTE) are rare entities and account for <1% of all cardioembolic strokes [1]. These clinical entities have distinct characteristic appearances that may be identified on transthoracic and transesophageal echocardiography, but distinguishing one from another remains a challenge for clinicians. Echocardiography is a noninvasive, low-cost, and time-effective procedure that can diagnose valvular lesions even at the bedside. PFE is often found incidentally and does not usually cause any symptoms. However, some patients may experience symptoms such as stroke, heart attack, syncope, and heart failure, or may even die [2]. NBTE is simply defined as vegetation on the heart valves. It is often linked to various diseases such as cancer, systemic lupus erythematosus, idiopathic antiphospholipid syndrome, and rheumatoid arthritis [3]. Other reported cases had similar presentations/history of recurrent strokes but their echocardiography findings differed from ours [4]. Our case highlights the importance of careful consideration of the full range of differential diagnoses for cardioembolic stroke, and a discussion of the roles of adjunctive diagnostic modalities [i.e., cardiac MRI (CMR) and cardiac CT (CCT)] to differentiate between these entities. This report discusses the case of a 49-year-old woman who presented with an embolic stroke and NBTE of the mitral valve with echocardiographic appearances mimicking PFE.

## Case presentation

A 49-year-old female with a past medical history significant for obesity, asthma, and type 2 diabetes mellitus presented to the emergency department with headache, numbness in the lateral three digits of the right hand, nausea, vomiting, and chills of a day's duration. She had no prior history of migraine or other recurrent headaches. Her past history was negative for cardiac or neurological conditions, and she denied any chest pain, palpitations, shortness of breath, or other neurological symptoms.

Her labs on admission were significant for an elevated cardiac troponin of 0.46 ng/mL (normal value: <0.30 ng/mL) that went up to 1.14 ng/mL on the next day. CT head obtained at the time of admission was unremarkable. However, due to her persistent headache and continued numbness of the right-hand digits, we obtained a CT angiogram of the head on the following day, which revealed an acute left parietal white matter infarct. Subsequent MRI of the brain showed multiple bi-hemispheric ischemic infarcts, highly suggestive of thromboembolic events.

**Figure 1:**
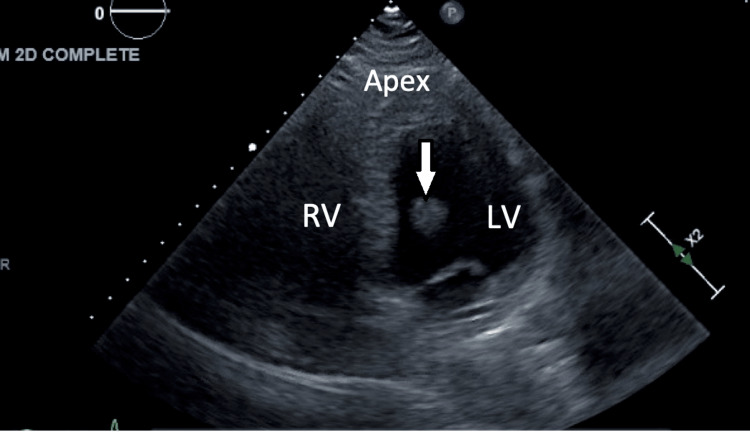
Transthoracic echocardiogram Two-chamber view on transthoracic echo revealing a mobile mass (white arrow) in the left ventricle (LV) RV: right ventricle

The workup for embolic stroke included a transthoracic echocardiogram (TTE) that revealed mildly impaired left ventricular (LV) systolic function with an ejection fraction (EF) of 45-50%, and a medium to large-sized mass attached to the mitral valve in the left ventricle, which was thought to represent a tumor, vegetation, or, less likely, a thrombus (Figure 1). In collaboration with the consulting neurologist, we started the patient on 81 mg aspirin daily and subcutaneous heparin for deep venous thrombosis (DVT) prophylaxis until the transesophageal echocardiogram (TEE) could be obtained. Workup was done to rule out autoimmune diseases and/or malignancies. Anti-nuclear antibody (ANA) was positive with the cytoplasmic pattern, but anticardiolipin, lupus anticoagulants, and antiphospholipid antibodies were negative. Anti-thrombin, factor V Leiden, and protein S activity were within normal limits. CT abdomen and pelvis did not show any masses or tumors.

**Figure 2:**
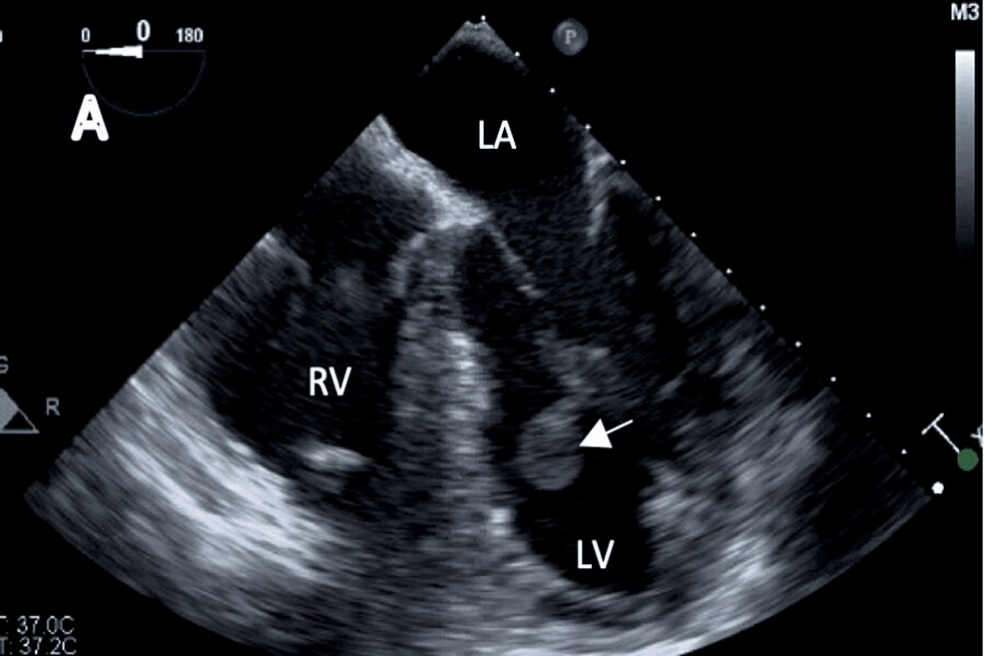
Transesophageal echocardiogram TEE images from various mid-esophageal angles, positioned to reveal a large pedunculated mass (white arrow) in the left ventricle (LV), attached to the chordae of the papillary muscle of the mitral valve LA: left atrium; RV: right ventricle

A TEE was obtained on hospital day three to better characterize the mass, and it revealed a 1.7 x 1.3 cm mass on the anterolateral papillary muscle, with an appearance consistent with PFE, and cardiothoracic surgery was consulted for resection (Figure 2). No anticoagulation was started at this time since we thought the patient had an embolic stroke due to the PFE diagnosed on the TEE. She was transferred to another hospital that has cardiothoracic surgeons in-house and is more familiar with such cases. Arranging transportation and waiting for bed availability took around three days.

**Figure 3:**
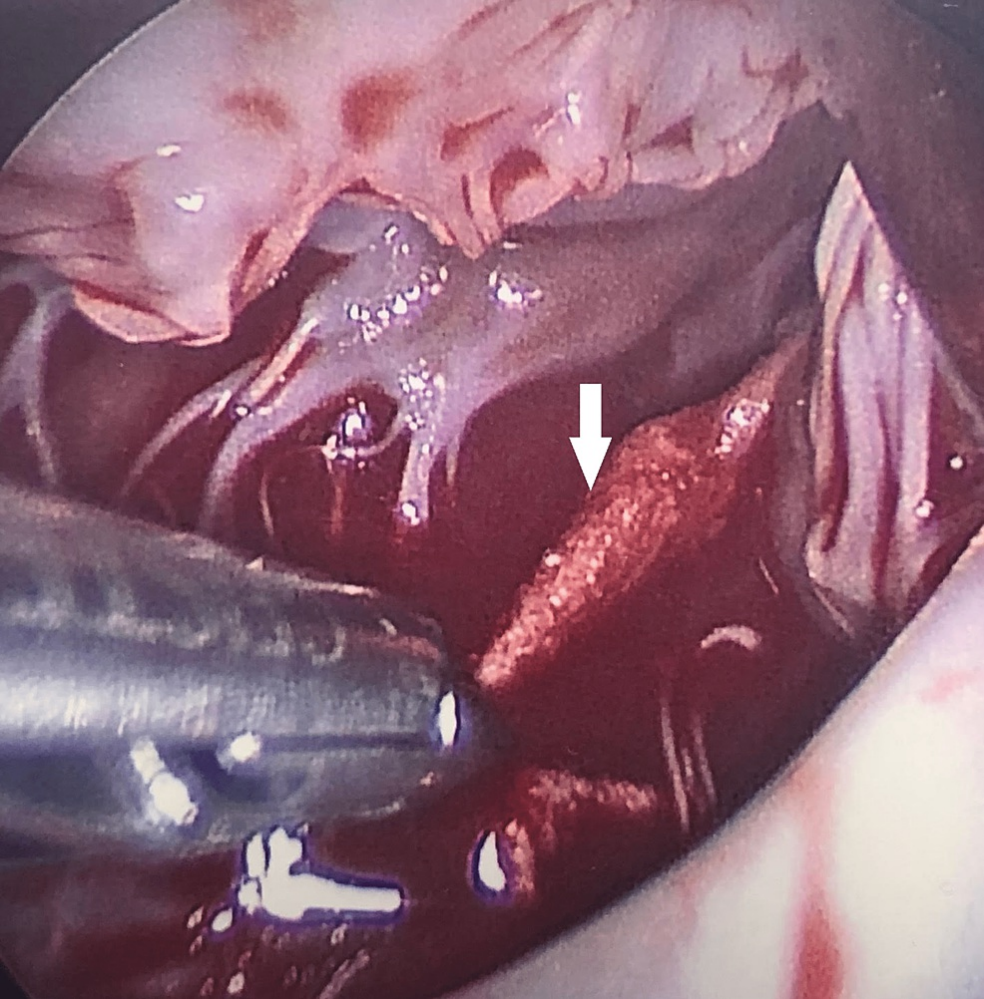
Intraoperative visualization of a large, friable mass (white arrow), seen under the anterior leaflet of the mitral valve and originating from the papillary muscle

**Figure 4:**
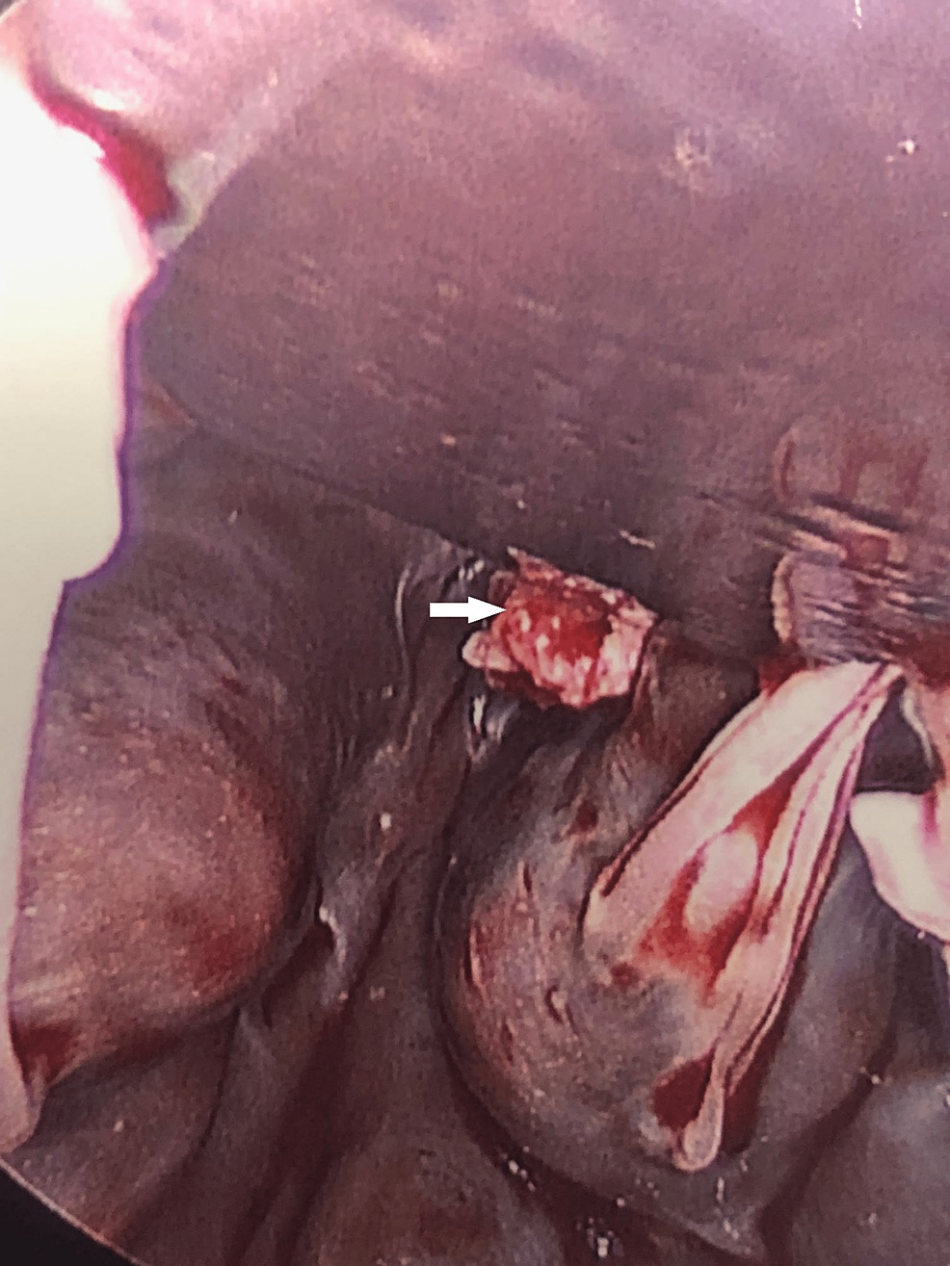
Stalk (white arrow) connecting the mass to the chordae of the papillary muscle

On hospital day seven, the patient underwent surgery, and a friable, pedunculated mass appearing to originate from the chordae of the papillary muscles was resected. In contrast with our anticipated finding of a fibroelastoma, the pathology report revealed a 1.7 x 1.4 x 0.2 cm organizing thrombus with abundant neutrophilic infiltration and no neoplastic proliferation (Figures 3, 4). She was subsequently discharged on apixaban with a planned follow-up in the outpatient setting.

## Discussion

NBTE is a rare disease and echocardiography is the initial diagnostic modality in case of clinical suspicion for NBTE; however, to establish a definitive diagnosis, microbiology and histopathology may be required. Around 13.7 to 17 million people experience a stroke each year globally [5,6]. Cardioembolic stroke accounts for 14-30% of all cerebral infarctions and <1% of these result from either PFE or NBTE [7,8]; we focus on these two conditions as well as the diagnostic challenges faced by clinicians to distinguish between these two very different clinical entities, which may require very different interventions in terms of magnitude. One is often safely managed with conservative medical therapy and systemic anticoagulation, while the other, in the context of embolic stroke, is an indication for open heart surgery [9].

PFEs are the second most common primary cardiac tumor in adults [10,11]. PFE's characteristic appearance on a two-dimensional echocardiogram is an independently mobile, pedunculated mass with a “speckled pattern” along the edge, ranging from 2 to 40 mm in size [2]. The size, shape, location, and appearance of the mass as seen on echo in our case were all consistent with the diagnosis of fibroelastoma. In the context of multiple embolic events, surgical intervention was indicated and, as such, elected as the treatment of choice without consideration of further imaging or another diagnostic testing as it was determined that regardless of etiology, given the high-risk features for a future embolic event, the additional information provided by adjunctive tests or imaging would not alter the course of management [12].

NBTE is a rare condition that has been reported in individuals of all age groups but is most common between the fourth and eighth decades of life and is often only discovered on postmortem examinations [13]. NBTE most commonly occurs in patients with advanced malignancy and those with systemic lupus erythematosus, with fewer cases identified in patients with inflammatory conditions such as antiphospholipid syndrome, rheumatic heart disease, rheumatoid arthritis, and burns [2], none of which were present in our patient. The vegetations typically consist of thrombi interwoven with strands of fibrin, immune complexes, and mononuclear cells. Stroke is the presenting complaint in 60% of cases [14]. Treatment of NBTE most often consists of systemic anticoagulation and therapy directed at treating the underlying malignancy or associated conditions [15]. Surgery is performed only in rare cases, mostly for select patients with embolic stroke in the presence of a large, highly mobile mass with a high risk of recurrent embolization [9].

Two-dimensional TTE is typically recommended as part of the initial evaluation of patients with suspicion of intracardiac masses. In the case of NBTE, vegetations are frequently left-sided and involve the mitral valve in 66% of cases [16]. One important limitation of echocardiography is its inability to distinguish vegetation due to thrombus from those due to infection, and in our case, there was ambiguity in distinguishing a thrombus from a tumor. Valvular vegetations are sometimes represented on echo by mobile, filamentous echo densities, which are thought to represent fibrin strands [17]. In the previously reported cases, an echocardiogram was accurate in diagnosing NBTE. However, in our case, echocardiogram results initially suggested PFE, which was later determined to be incorrect, and the patient was ultimately diagnosed with NBTE as per the histopathology report [4]. Negative TTE should be followed up with a TEE, which is almost 100% sensitive for the detection of vegetation, compared with the 63% sensitivity with TTE, even though it was unable to distinguish thrombus from the tumor in our case [18].

While this case presented a high-risk lesion with a clear indication for surgical resection, NBTE and PFE can present with lesions of widely varying size and degrees of risk for future events, and in such lesions, additional early diagnostic workup may serve to spare select patients from surgical intervention. Both CMR and CCT can provide high-resolution, noninvasive images of the heart. CMR is often preferred, as T1- and T2-weighted images can provide additional insights into the microenvironment of a tumor, which may be helpful in better characterizing masses of uncertain etiology [19,20].

## Conclusions

While echocardiography may aid in the differentiation of endocardial and valvular lesions, a conclusive diagnosis can only be reached through the utilization of microbiology and histopathology. Early utilization of advanced imaging techniques such as CCT or CMR may assist in identifying select cases that are at lower risk for subsequent embolic events, in which surgical intervention may safely be avoided.
